# Do patients with type 2 diabetes have impaired hip bone microstructure? A study using 3D modeling of hip dual-energy X-ray absorptiometry

**DOI:** 10.3389/fendo.2022.1069224

**Published:** 2023-01-09

**Authors:** Esther Ubago-Guisado, Enrique Moratalla-Aranda, Sheila González-Salvatierra, José J. Gil-Cosano, Beatriz García-Fontana, Cristina García-Fontana, Luis Gracia-Marco, Manuel Muñoz-Torres

**Affiliations:** ^1^Escuela Andaluza de Salud Pública (EASP), Granada, Spain; ^2^Instituto de Investigación Biosanitaria ibs.GRANADA, Granada, Spain; ^3^Epidemiology and Control of Chronic Diseases, CIBER of Epidemiology and Public Health (CIBERESP), Madrid, Spain; ^4^Department of Nuclear Medicine, University Hospital Clínico San Cecilio, Granada, Spain; ^5^Department of Medicine, University of Granada, Granada, Spain; ^6^Fundación para la Investigación Biosanitaria de Andalucía Oriental (FIBAO), Granada, Spain; ^7^PROFITH “PROmoting FITness and Health through Physical Activity”, Research Group, Sport and Health University Research Institute (iMUDS), Departament of Physical Education and Sport, Faculty of Sport Sciences, University of Granada, Granada, Spain; ^8^Department of Communication and Education, Universidad Loyola Andalucía, Dos Hermanas (Sevilla), Spain; ^9^Endocrinology and Nutrition Unit, University Hospital Clínico San Cecilio, Granada, Spain; ^10^CIBER de Fragilidad y Envejecimiento Saludable (CIBERFES), Instituto de Salud Carlos III, Madrid, Spain; ^11^Centro de Investigación Biomédica en Red Fisiopatología de la Obesidad y Nutrición (CIBERobn), Instituto de Salud Carlos III, Madrid, Spain

**Keywords:** type 2 diabetes mellitus, 3D-DXA, bone modelling, bone remodeling, bone QCT/microCT

## Abstract

**Aim:**

Patients with type 2 diabetes (T2DM) have more risk of bone fractures. However, areal bone mineral density (aBMD) by conventional dual-energy x-ray absorptiometry (DXA) is not useful for identifying this risk. This study aims to evaluate 3D-DXA parameters determining the cortical and trabecular compartments in patients with T2DM compared to non-diabetic subjects and to identify their determinants.

**Materials and methods:**

Case-control study in 111 T2DM patients (65.4 ± 7.6 years old) and 134 non-diabetic controls (64.7 ± 8.6-year-old). DXA, 3D-DXA modelling *via* 3D-Shaper software and trabecular bone score (TBS) were used to obtain aBMD, cortical and trabecular parameters, and lumbar spine microarchitecture, respectively. In addition, biochemical markers as 25-hydroxyvitamin d, type I procollagen N-terminal propeptide (P1NP), C-terminal telopeptide of type I collagen (CTX), and glycated haemoglobin (HbA1c) were analysed.

**Results:**

Mean-adjusted values showed higher aBMD (5.4%-7.7%, ES: 0.33-0.53) and 3D-DXA parameters (4.1%-10.3%, ES: 0.42-0.68) in the T2DM group compared with the control group. However, TBS was lower in the T2DM group compared to the control group (-14.7%, ES: 1.18). In addition, sex (β = 0.272 to 0.316) and body mass index (BMI) (β = 0.236 to 0.455) were the most consistent and positive predictors of aBMD (p ≤ 0.01). BMI and P1NP were negative predictors of TBS (β = -0.530 and -0.254, respectively, p ≤ 0.01), while CTX was a positive one (β = 0.226, p=0.02). Finally, BMI was consistently the strongest positive predictor of 3D-DXA parameters (β = 0.240 to 0.442, p<0.05).

**Conclusion:**

Patients with T2DM present higher bone mass measured both by conventional DXA and 3D-DXA, suggesting that 3D-DXA technology is not capable of identifying alterations in bone structure in this population. Moreover, BMI was the most consistent determinant in all bone outcomes.

## 1 Introduction

Patients with type 2 diabetes (T2DM) have increased bone fragility, which explains the increased risk of bone fractures in this population (1). However, areal bone mineral density (aBMD), as assessed by conventional dual-energy x-ray absorptiometry (DXA), is not useful for identifying this risk (2). In fact, most studies have shown that aBMD values at different sites (vertebral or femoral) are increased in these subjects (3). Elevated aBMD, in particular at the hip, is likely due to the higher body mass index (BMI) of people with T2DM. For this reason, there is great interest in having useful tools in clinical practice to identify patients with T2DM and high fracture risk.

The mechanisms that promote bone fragility in patients with T2DM are complex and poorly understood. The classic complications of T2DM (retinopathy, nephropathy and cardiovascular disease) and an increased likelihood of falls may explain part of the increased risk of fractures (4). However, there are data indicating that impairment in some specific bone parameters is a substantial cause of this bone fragility (5). Using complex techniques such as high-resolution peripheral quantitative computed tomography (HR-pQCT) or *in vivo* microindentation, it has been reported that patients with T2DM have an increased cortical porosity and a deficit in bone material properties (6). All these data suggest a deterioration of what we know as bone quality. However, these findings are not absolutely consistent and the availability of these techniques is very limited in clinical setting (7).

The trabecular bone score (TBS) is a simple method that estimates trabecular microstructure from DXA images of the lumbar spine (8). The technique is based on grey-level analyses of two-dimensional projection images from a DXA lumbar spine scan providing information on spinal trabecular microarchitecture and fracture risk independent of aBMD (9). A decrease in TBS values has been described in patients with T2DM, which is related to an increased risk of fracture (10). The finding of degraded trabecular microarchitecture is in contradiction with the studies performed with HR-pQCT where the trabecular component is preserved (11). Other limitations of TBS are that it can only be performed in the lumbar spine (not in the hip) and that its results may be a reflection of the abdominal obesity that characterizes these patients.

In recent years, 3D modeling methods have been developed to evaluate volumetric BMD (vBMD) as well as the cortical thickness, among other parameters, and differentiate trabecular and cortical compartments at the proximal femur from conventional DXA scans (12). To our knowledge, no studies have been published in patients with T2DM using this technique.

The main purpose of our study was to evaluate 3D-DXA parameters determining the cortical and trabecular compartments in patients with T2DM compared to non-diabetic subjects and to identify their determinants.

## 2 Material and methods

### 2.1 Study design and participants

This case-control study included a total of 245 participants, of whom 111 were patients with T2DM (43.2% females, 65.4 ± 7.6 years old), and 134 were non-diabetic controls (48.5% females, 64.7 ± 8.6-year-old). The recruitment was carried out from 2017 to 2018 in the Endocrinology and Nutrition Unit of the University Hospital Clínico San Cecilio of Granada (Spain). Diagnosis of T2DM was according to American Diabetes Association Criteria 2017. The inclusion criteria were as follows: Caucasians, free-living, ages from 35–65 years old, and normal values for their blood count, hepatic function, calcium, phosphorus and parathyroid hormone. Exclusion criteria included an estimated glomerular filtration rate (eGFR) < 60 mL/min/1.73m^2^ (serum creatinine was measured and eGFR was calculated using the CKD-EPI equation), the diagnosis of metabolic bone disease other than osteoporosis, a chronic disease known to affect bone metabolism, such as hyperparathyroidism, rheumatoid arthritis, hepatic and renal chronic diseases, and active neoplastic diseases as well as hormone replacement therapy, and glucocorticoid or osteoporosis therapy. All the T2DM patients were on oral anti diabetic treatment. No patients were treated with thiazolidinediones or SGLT2 inhibitors. The control group included apparently healthy subjects that were matched by sex and age. They were recruited from the general community in the same time window and ensuring they did not meet the exclusion criteria. Moreover, subjects with clinical fractures or significant cardiovascular disease were excluded.

This study was conducted with the approval of the Ethics Committee of the University Hospital Clínico San Cecilio of Granada in accordance with the Declaration of Helsinki by the World Medical Association (Project ID: 0858-N-17, Research Ethics Committee of Granada Center on 26 April 2017). Written informed consent was obtained from all participants.

### 2.2 Anthropometric measures

Height (cm) and weight (kg) were measured with a stadiometer (SECA 225 and 220, Hamburg, Germany), and an electronic scale (SECA 861 and 760, Hamburg, Germany), respectively. BMI was calculated as weight (kg)/height (m^2^).

### 2.3 Clinical evaluation

The presence of microvascular diseases (retinopathy, nephropathy or neuropathy) and cardiovascular disease (CVD) was obtained from clinical records and categorized (Yes/No). Moreover, the history of prevalent fractures was obtained by means of a clinical interview.

### 2.4 Areal bone mineral density and trabecular bone score assessment

The left hip and lumbar spine (L1-L4) were scanned using a Hologic QDR 4500 densitometer (Hologic Series Discovery QDR, Bedford, MA, US) to measure aBMD at the total hip, femoral neck, and lumbar spine. All DXA scans and analyses were performed by the same trained operator according to the recommendations from the International Society of Clinical Densitometry (13). A spine phantom was used to calibrate the DXA equipment on a daily basis. The coefficients of variation within our laboratory were 1.5%, 1.8%, and 1.5% for total hip, femoral neck and lumbar spine, respectively.

The latest available version of TBS iNsight (version 3.0.2.0, Medimaps, Merignac, France) was used to measure TBS at the lumbar spine based on a grey-level analysis of the DXA images (14). TBS was calculated as the mean value of the individual measurements for vertebrae L1−L4, with a coefficient of variation of 1.82%.

### 2.5 3D-DXA modelling

The 3D-Shaper software (version 2.2, Galgo Medical, Barcelona, Spain) was used to assess the cortex, femoral shape and trabecular macrostructure of the proximal femur from hip DXA scans of the T2DM and control groups. Detailed information about the modelling method has been published previously (15).

The cortical thickness (mm), the cortical surface BMD (sBMD, mg/cm^2^) and the volumetric (vBMD, mg/cm^3^) values of the cortical, trabecular and integral bone compartments of the total femur were computed. The cortical thickness and density are computed by fitting a mathematical function to the density profile computed along the normal vector at each node of the proximal femur surface mesh. The trabecular vBMD measures the density of the trabecular compartment. The cortical sBMD at each vertex of the femoral surface mesh was computed as the product between cortical thickness and the cortical vBMD along its thickness. The integral compartment is measured by the integral vBMD, which is the union of the cortical and trabecular compartments.

The correlations between 3D-DXA and QCT for the integral vBMD, cortical vBMD, trabecular vBMD and cortical thickness were 0.95, 0.93, 0.86 and 0.91, respectively. The coefficients of variation for the cortical sBMD, trabecular vBMD, cortical vBMD and cortical thickness were 1.5%, 4.5%, 1.7% and 1.5%, respectively.

### 2.6 Biochemical markers

Samples of venous blood were taken in the morning after fasting overnight. Serum samples were stored at −80 °C until they were analysed at the Clinical Analysis Unit of the University Hospital Clínico San Cecilio of Granada. The 25-hydroxyvitamin D [25(OH)D, ng/ml], type I procollagen N-terminal propeptide (P1NP, ng/ml), C-terminal telopeptide of type I collagen (CTX, ng/mL) and glycated haemoglobin (HbA1c, %) were obtained following standard laboratory protocols. More specifically, the 25(OH)D was determined with the two-site immunoassay (Roche Diagnostics SL, Barcelona, Spain). Bone turnover markers were measured as follows: CTX by enzyme immunoassay (Elecsys b-CrossLaps; Roche Diagnostics, Basel, Switzerland), and P1NP was analysed by immunoassay on an autoanalyzer COBAS 601 (Roche, Spain). Finally, HbA1c was determined using high-performance liquid chromatography (ADAMS A1c, HA-8160; Menarini, Florence, Italy). The intra- and inter-assay precision coefficients of variation were 6.9% and 7.2% for 25(OH)D; 2.0% and 2.9% for CTX and; 5.1% and 6.5% por P1NP.

### 2.7 Statistical analysis

Data were analysed using SPSS IBM statistics (version 20 for Windows, Chicago, IL) and the significance level was set at p<0.05. The distribution of variables was checked using the Kolmogorov-Smirnov test, skewness and kurtosis values, visual check of the histograms, and Q-Q and box plots. Continuous variables were expressed as mean and standard deviation (SD) and categorical variables as absolute number and percentage.

Descriptive analyses comparing the T2DM and control groups were performed by independent samples *T*-test. Analysis of covariance (ANCOVA) was used to examine differences in the outcome variables (aBMD, 3D-DXA parameters and TBS) between the T2DM and control groups. Age, sex and BMI were used as covariates. The effect size (ES, Cohen’s *d*) is shown with the following interpretation: 0.2 is small, 0.5 is medium, and 0.8 is large. Percentages of difference between groups were used to quantify the magnitude of the differences. The 3D spatial distribution of the differences (T2DM vs. controls) in the cortical bone (cortical sBMD, cortical vBMD and cortical thickness) is also shown.

Finally, multiple linear regression analyses were used to examine the contribution in the bone parameters of sex, age, BMI, time from diagnosis, 25(OH)D, P1NP, CTX, HbA1c, CVD, microvascular disease and fracture. The selection of the predictors was based on their relationship with bone outcomes (1, 5). All predictors were entered into the regression models simultaneously. The standardized regression coefficients (β) were reported and the squared semi-partial correlation coefficients (sr^2^) were used to determine the contribution of each predictor in the overall variance of the model after removing shared contributions with other predictors.

## 3 Results

**Table 1** shows descriptive characteristics of the participants in the T2DM and control groups. The proportion of males and females was similar in both groups. Both groups were comparable for age and height, but weight and BMI were higher in the T2DM group (all p<0.001). Crude aBMD and femoral 3D-DXA parameters were higher in the T2DM group compared to the control group (all p<0.05), but TBS was lower (p<0.001). In addition, 25(OH)D, P1NP, and CTX were lower in the T2DM group compared to the controls (all p<0.01), while HbA1c was higher (p<0.001).

**Table 1:** Descriptive characteristics and differences in areal bone mineral density (aBMD), TBS (trabecular bone score), and 3D-DXA outcomes of the study groups.

	T2DM (n=111)		Controls (n=134)		p
Sex (n, %) Male	63 (56.8%)		69 (51.5%)		0.411
Female	48 (43.2%)		65 (48.5%)		
	Mean	SD	Mean	SD	
Age (years)	65.4	7.6	64.7	8.6	0.526
Height (cm)	164.9	9.1	163.1	9.7	0.132
Weight (kg)	86.1	14.5	74.2	15.0	**<0.001**
BMI (kg/m^2^)	31.6	4.5	27.9	5.0	**<0.001**
Prevalent fracture (n, %)	14 (12.6%)		–		–
Time from diagnosis (years)	19.9	9.0	–	–	–
aBMD (g/cm^2^)					
Total hip	1.052	0.175	0.921	0.166	**<0.001**
Femoral neck	0.822	0.159	0.748	0.128	**<0.001**
Femoral neck T-score	-0.621	1.230	-0.955	1.045	**0.027**
Lumbar spine	1.043	0.198	0.964	0.167	**0.001**
Lumbar spine T-score	-0.452	1.560	-1.015	1.425	**0.004**
**Femoral 3D-DXA**					
Cortical sBMD (mg/cm^2^)	179.73	27.01	159.52	26.51	**<0.001**
Trabecular vBMD (mg/cm^3^)	190.17	51.09	162.84	41.73	**<0.001**
Cortical vBMD (mg/cm^3^)	871.36	64.62	819.28	74.82	**<0.001**
Integral vBMD (mg/cm^3^)	338.89	62.84	299.80	58.24	**<0.001**
Cortical thickness (mm)	2.12	0.29	1.93	0.19	**<0.001**
**TBS**	1.074	0.187	1.291	0.110	**<0.001**
Biochemical markers					
25(OH)D (ng/ml)	20.35	7.83	25.83	8.57	**<0.001**
P1NP (ng/ml)	37.50	15.10	44.11	20.88	**0.007**
CTX (ng/mL)	0.20	0.15	0.36	0.21	**<0.001**
HbA1c (%)	7.89	1.40	5.59	0.32	**<0.001**
eGFR (mL/min/1.73 m^2^)	84.28	19.74	82.72	16.03	0.503
Cardiovascular risk factors					
Cardiovascular disease (n, %)	42 (37.8%)		–		–
Microvascular disease (n, %)	38 (34.2%)		–		–

Values presented as mean and SD (for continuous variables), or absolute number and percentage (for categorical variables).

Boldface denotes significant values (p<0.05).

T2DM, type 2 diabetes mellitus; BMI, body mass index; aBMD, areal bone mineral density; sBMD, surface bone mineral density; vBMD, volumetric bone mineral density; TBS, trabecular bone score; eGFR, estimated glomerular filtration rate; HbA1c, glycated haemoglobin; 25(OH)D, 25-hidroxivitamin D; P1NP, type I procollagen N-terminal propeptide; CTX, C-terminal telopeptide of type I collagen.

**Figure 1:**
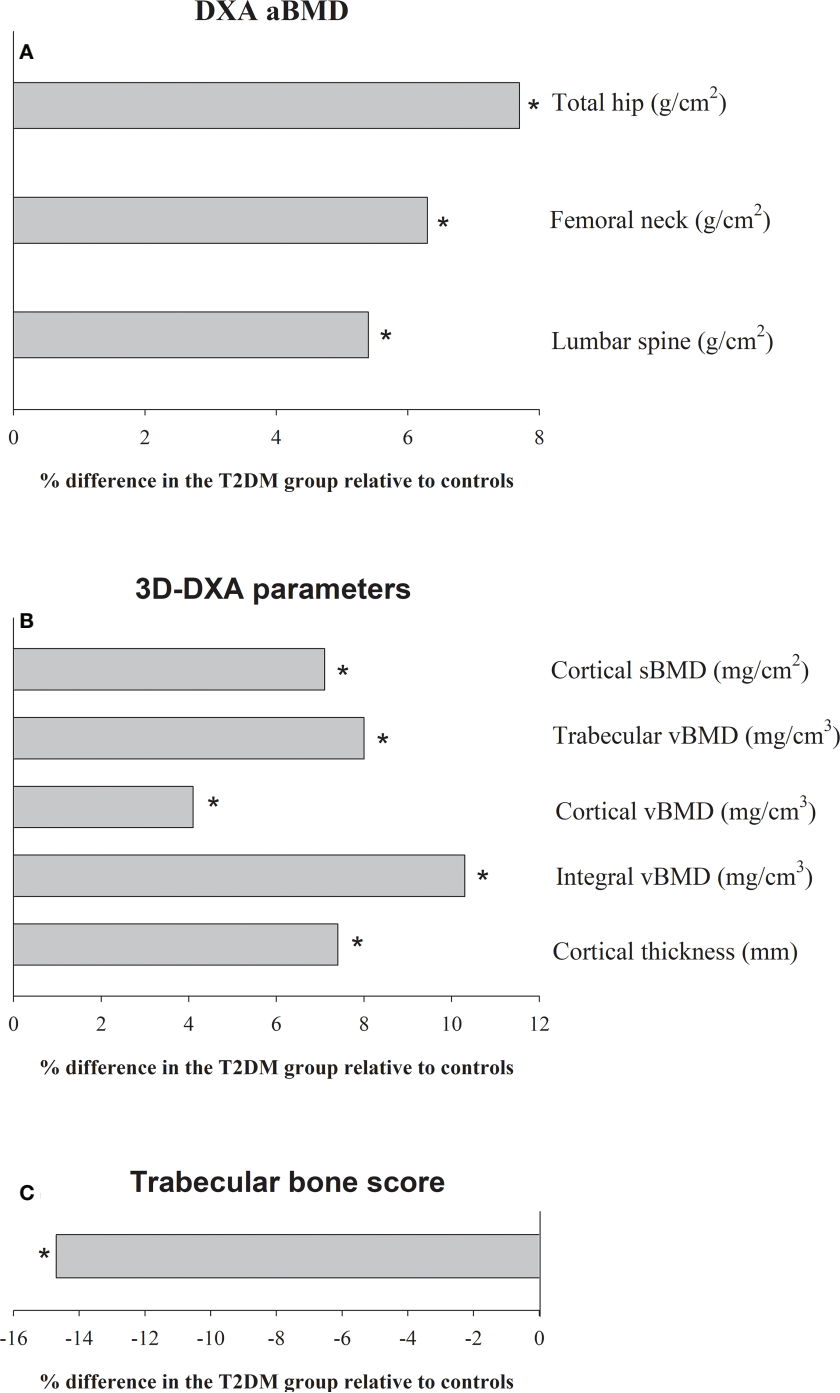
Adjusted differences (%) (by age, sex and body mass index) in **(A)** the areal bone mineral density (aBMD), **(B)** 3D-DXA parameters, and **(C)** trabecular bone score (TBS), between patients with T2DM (n = 111) and the control group (n = 134). * denote significant differences (p<0.05).

**Table 2** shows mean-adjusted differences in aBMD and 3D-DXA parameters between groups. aBMD was higher in the T2DM group compared with the control group at all sites: total hip (p<0.001, ES=0.53), femoral neck (p=0.01, ES=0.37) and lumbar spine (p=0.02, ES=0.33). Specifically, the difference was 7.7%, 6.3% and 5.4% at the total hip, femoral neck and lumbar spine, respectively (**Figure 1A**). 3D-DXA parameters were higher in the T2DM group compared to controls at all sites: cortical sBMD (p<0.001, ES=0.53), trabecular vBMD (p=0.002, ES=0.42), cortical vBMD (p<0.001, ES=0.52), integral vBMD (p=0.001, ES=0.45) and cortical thickness (p<0.001, ES=0.68). Specifically, the difference was 7.4%, 10.3%, 4.1%, 8.0%, and 7.1% for the cortical sBMD, trabecular vBMD, cortical vBMD, integral vBMD and cortical thickness, respectively (**Figure 1B**). Finally, TBS was lower in the T2DM group than in the control group (p<0.001, ES=1.18) with a percentage difference of -14.7% (**Figure 1C**).

**Table 2:** Mean-adjusted (by age, sex and body mass index) differences in areal bone mineral density (aBMD), TBS (trabecular bone score), and 3D-DXA parameters between the study groups.

	T2DM (n=111)		Controls (n=134)		p	Effect size^¥^
	Mean	95% CI	Mean	95% CI		
aBMD (g/cm^2^)						
Total hip	1.024	0.996 - 1.052	0.945	0.919 - 0.970	**<0.001**	0.53
Femoral neck	0.809	0.784 - 0.835	0.758	0.735 - 0.781	**0.005**	0.37
Lumbar spine	1.030	0.998 - 1.062	0.974	0.945 - 1.004	**0.016**	0.33
Femoral 3D-DXA						
Cortical sBMD (mg/cm^2^)	175.76	171.15 - 180.36	162.82	158.66 - 166.98	**<0.001**	0.53
Trabecular vBMD (mg/cm^3^)	185.66	177.16 - 194.14	166.58	158.91 - 174.25	**0.002**	0.42
Cortical vBMD (mg/cm^3^)	862.34	849.52 - 875.16	826.75	815.16 - 838.34	**<0.001**	0.52
Integral vBMD (mg/cm^3^)	332.03	320.92 - 343.15	305.47	295.43 - 315.52	**0.001**	0.45
Cortical thickness (mm)	2.10	2.05 - 2.14	1.95	1.91 - 1.99	**<0.001**	0.68
**TBS**	1.104	1.078 – 1.129	1.266	1.243 – 1.289	**<0.001**	1.18

Values presented as mean and standard error.

Boldface denotes significant values (p<0.05).

T2DM, type 2 diabetes mellitus; aBMD, areal bone mineral density; sBMD, surface bone mineral density; vBMD, volumetric bone mineral density; TBS, trabecular bone score.

^¥^ The interpretation of ES is: 0.2 (small), 0.5 (medium) and 0.8 (large).

**Figure 2:**
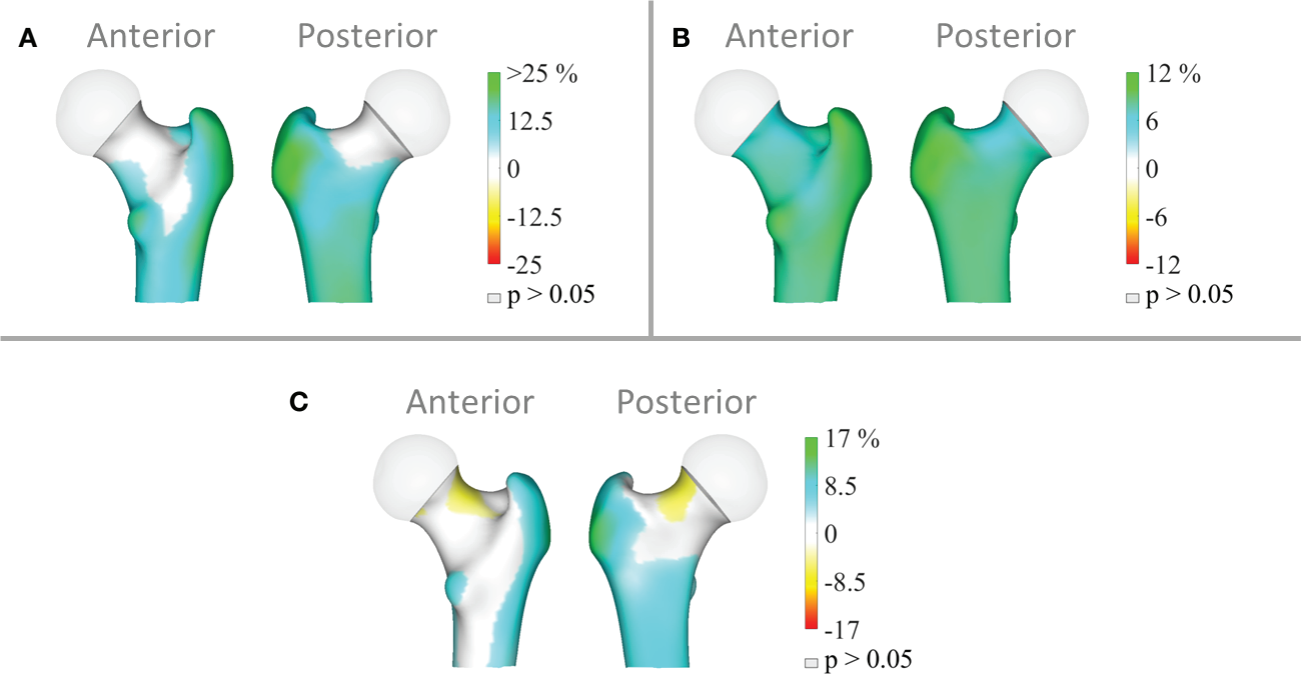
Distribution of the average differences (p<0.05) in **(A)** cortical surface bone mineral density (cortical sBMD), **(B)** cortical volumetric bone mineral density (cortical vBMD) and **(C)** cortical thickness of the total femur region in the T2DM group (n=111) relative to the controls (n=134). Regions with non-significant changes (p>0.05) are shown in grey.

3D mapping showing the anatomical distribution of differences between groups in the cortical compartment and indicates that the T2DM group had higher cortical sBMD at the trochanter, intertrochanteric area and subtrochanteric area (**Figure 2A**); higher cortical vBMD at the whole femoral region (femoral neck, trochanter, intertrochanteric area and subtrochanteric area) (**Figure 2B**); and higher cortical thickness at the trochanter and subtrochanteric area (**Figure 2C**) (all p<0.05). Surprisingly, a small region of the femoral neck shows significantly lower cortical thickness in the T2DM group (**Figure 2C**).

**Tables 3**, **4** present multiple linear regression models for bone parameters (aBMD, TBS, and femoral 3D-DXA) in the T2DM group. These models significantly explained 20% - 36% of the variance in the aBMD parameters (**Table 3**), 39% of the variance in TBS (**Table 3**), and 21% - 33% of the variance in the femoral 3D-DXA parameters (**Table 4**).

**Table 3:** Multiple regression models for bone parameters (aBMD and TBS) in patients with T2DM.

	Predictors	β STD	sr^2^ values	P values		Predictors	β STD	sr^2^ values	P values
**Total hip aBMD** **(R^2 =^ 0.36)**	Sex Age BMI Time from diagnosis 25(OH)D P1NP CTX HbA1c CVD Microvascular disease Fracture	0.272 -0.081 0.455 0.146 -0.104 -0.181 -0.052 -0.093 0.005 -0.153 -0.012	0.058 0.005 0.192 0.015 0.009 0.028 0.002 0.007 <0.001 0.015 <0.001	**0.005** 0.405 **<0.001** 0.151 0.251 0.050 0.576 0.311 0.958 0.149 0.891	**Lumbar spine aBMD** **(R^2 =^ 0.20)**	Sex Age BMI Time from diagnosis 25(OH)D P1NP CTX HbA1c CVD Microvascular disease Fracture	0.316 -0.152 0.117 0.113 0.021 0.002 -0.102 -0.008 0.126 -0.091 0.062	0.078 0.018 0.013 0.009 <0.001 <0.001 0.008 0.007 0.011 0.005 0.004	**0.004** 0.161 0.234 0.319 0.831 0.984 0.331 0.390 0.258 0.437 0.520
**Femoral neck aBMD** **(R^2 =^ 0.24)**	Sex Age BMI Time from diagnosis 25(OH)D P1NP CTX HbA1c CVD Microvascular disease Fracture	0.203 -0.280 0.236 0.197 0.074 -0.109 -0.014 -0.005 0.127 -0.244 0.033	0.032 0.060 0.052 0.027 0.005 0.010 <0.001 <0.001 0.012 0.038 0.001	0.051 **0.009** **0.014** 0.075 0.452 0.274 0.887 0.956 0.242 **0.035** 0.722	**TBS** **(R^2 =^ 0.39)**	Sex Age BMI Time from diagnosis 25(OH)D P1NP CTX HbA1c CVD Microvascular disease Fracture	0.074 -0.154 -0.530 -0.052 0.044 -0.254 0.226 0.026 -0.047 -0.004 0.018	0.004 0.018 0.261 0.002 0.002 0.055 0.041 0.001 0.002 <0.001 <0.001	0.426 0.106 **<0.001** 0.601 0.615 **0.005** **0.015** 0.766 0.626 0.966 0.831

Boldface denotes significant values (p<0.05).

T2DM, type 2 diabetes mellitus; β, standardised regression coefficient; sr^2^, squared semi-partial correlation coefficients; aBMD, areal bone mineral density; TBS, trabecular bone score; BMI, body mass index; 25(OH)D, 25-hidroxivitamin D; P1NP, type I procollagen N-terminal propeptide; CTX, C-terminal telopeptide of type I collagen; HbA1c, glycated haemoglobin; CVD, cardiovascular disease.

**Table 4:** Multiple regression models for bone parameters (femoral 3D-DXA) in patients with T2DM.

	Predictors	β STD	sr^2^ values	P values		Predictors	β STD	sr^2^ values	P values
**Cortical sBMD** **(R^2 =^ 0.33)**	Sex Age BMI Time from diagnosis 25(OH)D P1NP CTX HbA1c CVD Microvascular disease Fracture	0.239 -0.178 0.433 0.198 -0.010 -0.058 -0.094 0.015 0.045 -0.266 -0.026	0.045 0.024 0.174 0.027 <0.001 0.003 0.007 <0.001 0.001 0.045 0.001	**0.016** 0.074 **<0.001** 0.058 0.917 0.532 0.327 0.875 0.655 **0.015** 0.769	**Integral vBMD** **(R^2 =^ 0.27)**	Sex Age BMI Time from diagnosis 25(OH)D P1NP CTX HbA1c CVD Microvascular disease Fracture	-0.056 -0.258 0.362 0.099 -0.035 -0.128 -0.150 -0.029 -0.017 -0.119 -0.008	0.002 0.050 0.122 0.007 0.001 0.014 0.018 0.001 <0.001 0.009 <0.001	0.583 **0.014** **<0.001** 0.362 0.715 0.192 0.135 0.766 0.873 0.292 0.931
**Trabecular vBMD** **(R^2 =^ 0.28)**	Sex Age BMI Time from diagnosis 25(OH)D P1NP CTX HbA1c CVD Microvascular disease Fracture	-0.009 -0.294 0.335 0.043 -0.020 -0.157 -0.188 -0.028 -0.011 -0.081 0.006	<0.001 0.066 0.104 0.001 <0.001 0.021 0.029 0.001 <0.001 0.004 <0.001	0.931 **0.005** **<0.001** 0.691 0.834 0.107 0.060 0.771 0.916 0.469 0.950	**Cortical thickness** **(R^2 =^ 0.21)**	Sex Age BMI Time from diagnosis 25(OH)D P1NP CTX HbA1c CVD Microvascular disease Fracture	0.123 -0.235 0.240 0.324 0.012 -0.042 0.020 0.164 0.012 -0.088 -0.028	0.008 0.042 0.054 0.073 <0.0010.002 <0.0010.023 <0.001 0.005 <0.001	0.329 **0.031** **0.015** **0.005** 0.902 0.677 0.850 0.107 0.912 0.450 0.771
**Cortical vBMD** **(R^2 =^ 0.27)**	Sex Age BMI Time from diagnosis 25(OH)D P1NP CTX HbA1c CVD Microvascular disease Fracture	0.064 -0.121 0.442 0.157 -0.078 -0.091 -0.101 -0.018 -0.023 -0.167 0.009	0.003 0.011 0.181 0.017 0.005 0.007 0.008 <0.001 <0.001 0.018 <0.001	0.525 0.240 **<0.001** 0.145 0.415 0.353 0.311 0.854 0.831 0.139 0.924					

Boldface denotes significant values (p<0.05).

T2DM, type 2 diabetes mellitus; β, standardised regression coefficient; sr2, squared semi-partial correlation coefficients; aBMD, areal bone mineral density; TBS, trabecular bone score; BMI, body mass index; 25(OH)D, 25-hidroxivitamin D; P1NP, type I procollagen N-terminal propeptide; CTX, C-terminal telopeptide of type I collagen; HbA1c, glycated haemoglobin; CVD, cardiovascular disease.

Our findings depicted in **Table 3** show sex (e.g., being a man) and BMI as the most consistent and positive predictors of aBMD at all sites (β = 0.272 to 0.316 for sex, and β = 0.236 to 0.455 for BMI, p ≤ 0.01). Age and the presence of microvascular disease were negatively associated with femoral neck aBMD (β = -0.280 and -0.244, respectively, p<0.05). In addition, BMI and P1NP were negative predictors of TBS (β = -0.530 and -0.254, respectively, p ≤ 0.01), while CTX was a positive one (β = 0.226, p=0.02).

In **Table 4**, BMI was consistently the strongest positive predictor of 3D-DXA parameters at all sites (β = 0.240 to 0.442, p<0.05). Age was the second strongest and negative predictor for integral vBMD, trabecular vBMD, and cortical thickness (β = -0.294 to -0.235, p<0.05). Sex (e.g., being a man) was positively associated with cortical sBMD (β = 0.239, p=0.02), and the presence of microvascular disease was negatively associated (β = -0.266, p=0.01). Finally, time from diagnosis was positively associated with cortical thickness (β = 0.324, p=0.01).

## 4 Discussion

This is the first study to simultaneously examine the results of conventional DXA, 3D-DXA of the proximal femur and TBS of patients with T2DM and non-diabetic controls. In addition, we analysed the determinants of the bone parameters in patients with T2DM. The main findings of the present study are that T2DM patients showed significantly higher values in aBMD and 3D-DXA trabecular and cortical parameters compared with the control group. However, TBS was significantly lower in the T2DM group compared with the control group. In addition, while BMI was the most consistent predictor of all bone outcomes (DXA, 3D-DXA and TBS), sex (e.g., being a man) specifically determined aBMD outcomes and age specifically contributed to 3D-DXA parameters. Finally, other factors such as the presence of microvascular disease, bone remodelling markers (P1NP and CTX), and time from diagnosis also had a significant contribution to some specific bone parameters.

Our results in aBMD outcomes and TBS are supported by a recent comprehensive review in 2022 (1). Patients with T1DM or T2DM have a higher risk of fragility fracture compared with non-diabetic controls (1). However, a higher skeletal fragility and fracture risk in patients with T2DM is usually associated with increased aBMD values by DXA (16), a fact known as “the diabetic paradox of bone fragility” (17). Specifically, in a meta-analysis of 15 observational studies (2), the participants with T2DM had significantly higher BMD at the total hip, femoral neck, and lumbar spine compared with the controls. Similar results were recently published by Hayon-Ponce et al. (10) where participants with T2DM had lower TBS values despite having higher values in the aBMD lumbar spine compared to controls. Also, Indian woman with T2DM presented similar (lumbar spine) or higher (total hip and femoral neck) aBMD and lower TBS values compared with non-diabetic controls (18). Thus, TBS seems to be a more effective tool than aBMD from DXA to know the status of lumbar spine bone microarchitecture and to identify fracture risk in patients with diabetes (19, 20).

The analysis of bone microarchitecture degradation in patients with T2DM has raised great interest in recent years to explain bone fragility in these subjects. However, studies performed with HR-pQCT in peripheral bones (tibia and radius) have shown contradictory results. Whilst some studies have described deficits in the cortical compartment and, in particular, an increase in cortical porosity in patients with T2DM (6, 21–23) others have found no differences in cortical vBMD in subjects with or without diabetes (24–26) and no increased cortical porosity (26, 27). The explanation for these discrepant findings may be related to different factors such as the different demographic characteristics of the populations studied, the severity of microvascular or macrovascular complications, or that the scans are performed in peripheral bones and not in the hip. In fact, in the study by Shanbhogue VV et al. it is pointed out that deficits in cortical microstructure are only detected in a subgroup of patients with advanced microvascular complications (6). Our results showed that all the parameters obtained by the 3D-DXA technique at the hip were significantly increased in patients with T2DM. However, the 3D mapping showed a small region in the femoral neck where patients with T2DM had significantly lower cortical thickness than controls. We currently do not know the clinical significance of this finding but might partially explain the increased risk of femoral neck fractures in this population. Future longitudinal studies are required to confirm this finding.

On the other hand, studies analyzing *in vivo* alterations of bone material properties in patients with T2DM by microindentation indicate a consistent decrease in bone strength (7, 28, 29). In this regard, recent studies that determine Advanced Glycation End-Products by skin autofluorescence are a promising non-invasive technique that should be validated in future studies (30, 31). Similarly, reduced bone remodeling, such as the one found in our study, may result in the inability of the bone to repair micro damages which when propagated lead to bone fracture (32).

Regarding the determinants that best explain the variance in bone outcomes in patients with T2DM, we found that younger men with higher BMI had higher aBMD outcomes at all sites. Moreover, those without microvascular disease had higher aBMD at the femoral neck. For 3D-DXA parameters, younger patients (male and female) with higher BMI had higher values in cortical and trabecular parameters. Moreover, those without microvascular disease had higher cortical sBMD and those with lower concentrations of bone remodelling markers (PINP and CTX) had higher trabecular vBMD. Moreover, those whose T2DM was diagnosed long time ago had higher cortical thickness. Finally, younger patients (male and female) with lower BMI and PINP values (though higher for CTX) had higher TBS. Overall, the scientific literature has confirmed age as a strong determinant of bone mass, both in health and disease (33). An excess of BMI is often encountered in patients with T2DM and it is related to increased aBMD and 3D-DXA parameters. The divergent influence of BMI on aBMD/3D-DXA and TBS values requires special attention. Probably, the mechanical effect of overweight on the skeleton and the hyperinsulinism accompanying type 2 diabetes explain the aBMD and 3D-DXA results in hip (34). Conversely, increased abdominal adiposity influences the decrease in TBS described in these patients (10). Microvascular disease may explain alterations of bone microarchitecture and quality (35) to bone fragility in T2DM (36, 37), an effect that may be, to some extent, driven by insulin resistance (38). We identified a longer duration of T2DM as a determinant of higher cortical thickness. Conversely, a recent meta-analysis found increased risk of hip and non-vertebral fractures in subjects with longer diabetes duration (39), although the authors did not investigate the effect on aBMD. Our finding is in line with that reported by Oei et al. (40) comparing T2DM groups Vs. a non-diabetic group. They observed that the differences in cortical thickness were more evident at older ages. As suggested, the lack of cortical thinning could impair bone remodelling with consequent lack of compensatory bone expansion, suggesting an inefficacious reallocation of bone in T2DM (41).

This novel study has certain limitations and strengths that should be mentioned. The cross-sectional design does not allow investigating causality, just associations. Information on fracture prevalence was obtained from clinical interviews but not verified by radiographic exploration which could explain the low fracture prevalence (12.6%) in our sample. However, this is a novel study combining conventional DXA aBMD outcomes, cortical and trabecular 3D-DXA parameters and TBS in T2DM. The sample represents daily clinical practice in 2 homogenous groups.

In this case-control study we found higher bone mass in patients with T2DM measured both by conventional DXA and 3D-DXA, suggesting that 3D-DXA technology is not capable of identifying alterations in bone structure in this population. Moreover, BMI was the most consistent determinant in all bone outcomes. The contribution of the other predictors was site-specific. Future studies using other non-invasive techniques that measure bone quality are needed.

## Data availability statement

The datasets presented in this article are not readily available because restrictions apply to the availability of some or all data generated or analyzed during this study to preserve patient confidentiality. The corresponding author will on request detail the restrictions and any conditions under which access to some data may be provided. Requests to access the datasets should be directed to BG-F (bgfontana@fibao.es), CG-F (cgfontana@hotmail.com) and LG-M (lgracia@ugr.es).

## Ethics statement

The studies involving human participants were reviewed and approved by Ethics Committee of the University Hospital Clínico San Cecilio of Granada in accordance with the Declaration of Helsinki by the World Medical Association (Project ID: 0858-N-17, Research Ethics Committee of Granada Center on 26 April 2017). The patients/participants provided their written informed consent to participate in this study.

## Author contributions

Conceptualization: MM-T and LG-M. Methodology: SS, BG and CG-F. Formal analysis: LG-M, MM-T and EU-G. Investigation: EM-A, JG-C, SS, BG, CG-F and LG-M. Writing—original draft preparation: EM-A and EU-G. Writing—review and editing: SS, JG-C, BG, CG-F, LG-M and MM-T. All authors contributed to the article and approved the submitted version.
